# The Royal British Nurses' Association.—I

**Published:** 1916-06-24

**Authors:** 


### THE MATRONS' AND SISTERS' DEPARTMENT.

### THE ROYAL BRITISH NURSES' ASSOCIATION.—I.

The Royal British Nurses' Association was founded in 1887, and H.R.H. the Princess Christian of Schleswig-Holstein became its first President. To the Association is due the credit of having made the first great effort to remove from the ranks nurses who were inadequately trained or who were likely to prove a discredit to their fellow-workers and a hindrance to the advancement of nursing. Internal troubles, to which we have referred, and which ought never to have arisen, have prevented the governing body from carrying to a successful issue several schemes for improving the status and economical position of nurses, but, apart from anything else which it may have accomplished, nurses owe much to the Association, for it established the three years' standard of training. Steadily, since it was founded, hospital after hospital has adopted the three years' course, while a Select Committee of the House of Lords expressed the opinion that a three years' training should be the minimum one for a fully-qualified nurse. What the Association accomplished in this respect was not attained without persevering effort, and no small amount of opposition and misrepresentation had to be encountered.

In the early years of its history the British Nurses' Association made an unsuccessful application for registration under the Companies Act, and in 1892 it drew up a petition praying to be incorporated by Royal Charter. H.R.H. the President presented the petition in her own name, and it was heard before the Lords in the latter part of the same year, the case for the Association being supported by Sir Horace Davey, Mr. Muir Mackenzie, and the Hon. H. W. Cross, M.P. In May 1893 Her Royal Highness announced to the General Council of the Corporation that the Queen, acting on the advice of her Privy Council, had decided to grant the Association a Royal Charter of Incorporation. Such incorporation meant much, not only to members of the Association itself, but to all nurses trained in Great Britain and her Dominions, for it raised their calling to the dignity of a profession. There were, however, heavy charges incidental to obtaining the Charter. Also the successful opposition to the application for incorporation under the Companies Act and the unsuccessful opposition to the petition for incorporation by Royal Charter were only resisted at very heavy cost, so that, later, financial anxieties often handicapped the work of the Association.

The system of registration of nurses by the Corporation, carried out under powers conferred by the Charter, has always been very thorough. In every case, before the name of a nurse is placed on the Register, confidential reports are obtained from every institution where she has held an appointment, and the Association has powers to erase from its Register the name of any nurse who, after due inquiry, has shown herself unworthy to have her name inscribed thereon. The Association is the only nursing body which possesses powers to grant a diploma in nursing, and a few years ago it instituted an examination for this, but owing to the fact that the examiners are all more or less engaged in military work, and that nurses have now little time at their own disposal, it was decided to discontinue the examinations for the duration of the war.

(*To be continued.*)

